# Supplementary material for: Phenotypic analysis of catastrophic childhood epilepsy genes
Source: Commun Biol. 2021 Jun 3;4:680. doi: 10.1038/s42003-021-02221-y (PMC8175701; doi:10.1038/s42003-021-02221-y)
Supplement: Supplementary file 1 — Supplementary Information [file 42003_2021_2221_MOESM1_ESM.pdf]

Supplementary Table 1: Genes associated with an epilepsy phenotype that were considered for the Epilepsy Zebrafish Project

Human Gene	OMIM	Phenotype	Inheritance	Mechanism
SCN1A	182389	Epileptic encephalopathy, early infantile (Dravet syndrome)	Autosomal dominant	LOF
SCN1B	600235	Atrial fibrillation, familial, 13	Autosomal dominant	LOF
		Brugada syndrome 5		
		Cardiac conduction defect, nonspecific		
		Epilepsy, generalized, with febrile seizures plus, type 1		
		Epileptic encephalopathy, early infantile, 52	Autosomal dominant	
SCN8A	600702	Cognitive impairment with or without cerebellar ataxia	Autosomal dominant	LOF
		Epileptic encephalopathy, early infantile, 13	Autosomal dominant	
		Seizures, benign familial infantile, 5	Autosomal dominant	
SCN9A	603415	Epilepsy, generalized, with febrile seizures plus, type 7	Autosomal dominant	n/a
		Erythermalgia, primary	Autosomal dominant	
		Febrile seizures, familial, 3B	Autosomal dominant	
		HSAN2D, autosomal recessive	Autosomal recessive	
		Insensitivity to pain, congenital	Autosomal recessive	
		Paroxysmal extreme pain disorder,	Autosomal dominant	
		Small fiber neuropathy	Autosomal dominant	
		{Dravet syndrome, modifier of}		
KCNA2	176262	Epileptic encephalopathy, early infantile, 32	Autosomal dominant	LOF
KCNMA1	600150	Cerebellar atrophy, developmental delay, and seizures	Autosomal dominant	
		Paroxysmal nonkinesigenic dyskinesia, 3, with or without generalized epilepsy		
KCNQ3	602232	Seizures, benign neonatal, type 2	Autosomal dominant	
KCTD1	613420	Scalp-ear-nipple syndrome	Autosomal dominant	
GABRA1	137160	Epileptic encephalopathy, early infantile	Autosomal dominant	LOF
GABRB3	137192	Epileptic encephalopathy, early infantile, 43	Autosomal dominant	LOF
		{Epilepsy, childhood absence, susceptibility to, 5}		
GABRG2	137164	Epilepsy, generalized, with febrile seizures plus, type 3	Autosomal dominant	LOF
		Febrile seizures, familial, 8		
		{Epilepsy, childhood absence, susceptibility to, 2}		
GRIN1	138249	Mental retardation, autosomal dominant 8	n/a	n/a
GRIN2A	138253	Epilepsy, focal, with speech disorder and with or without mental retardation	Autosomal dominant	LOF
SLC13A5	608305	Epileptic encephalopathy, early infantile, 25	Autosomal recessive	LOF
SLC25A22	609302	Epileptic encephalopathy, early infantile, 3	Autosomal recessive	LOF
SLC2A1	138140	Dystonia 9	Autosomal dominant	LOF
		GLUT1 deficiency syndrome 1, infantile onset, severe	Autosomal recessive<br>Autosomal dominant	
		GLUT1 deficiency syndrome 2, childhood onset	Autosomal dominant	
		Stomatin-deficient cryohydrocytosis with neurologic defects	Autosomal dominant	
		{Epilepsy, idiopathic generalized, susceptibility to, 12}	Autosomal dominant	

SLC35A2	314375	Congenital disorder of glycosylation, type IIm	X-linked dominant; Somatic mosaicism	LOF
		Epileptic encephalopathy, early infantile, 22		
SLC6A1	137165	Myoclonic-atonic epilepsy	Autosomal dominant	LOF
DNM1	602377	Epileptic encephalopathy, early infantile, 31	Autosomal dominant	LOF
GOSR2	604027	Epilepsy, progressive myoclonic 6	Autosomal recessive	LOF
PRRT2	614386	Convulsions, familial infantile, with paroxysmal choreoathetosis	Autosomal dominant	LOF
		Episodic kinesigenic dyskinesia 1	Autosomal dominant	LOF
		Seizures, benign familial infantile, 2	Autosomal dominant	LOF
SPTAN1	182810	Epileptic encephalopathy, early infantile, 5	Autosomal dominant	
STX1B	601485	Generalized epilepsy with febrile seizures plus, type 9	Autosomal dominant	
STXBP1	602926	Epileptic encephalopathy, early infantile, 4	Autosomal dominant	
SYN1	313440	Epilepsy, X-linked, with variable learning disabilities and behavior disorders	X-linked recessive; X-linked dominant	
SYNGAP1	603384	Mental retardation, autosomal dominant 5	Autosomal dominant	
ARX	300382	Epileptic encephalopathy, early infantile, 1	X-linked recessive	LOF
		Hydranencephaly with abnormal genitalia	X-linked	
		Lissencephaly, X-linked 2	X-linked	
		Mental retardation, X-linked 29 and others	X-linked recessive	
		Partington syndrome	X-linked recessive	
		Proud syndrome	X-linked	
EEF1A2	602959	Epileptic encephalopathy, early infantile, 33	Autosomal dominant	n/a
		Mental retardation, autosomal dominant 38		
HNRNPU	602869	Epileptic encephalopathy, early infantile	Autosomal dominant	LOF
MEF2C	600662	Mental retardation, stereotypic movements, epilepsy, and/or cerebral malformations	Autosomal dominant	LOF
		Chromosome 5q14.3 deletion syndrome	Autosomal dominant	
PNKP	605610	Ataxia-oculomotor apraxia 4	Autosomal recessive	LOF
		Microcephaly, seizures, and developmental delay	Autosomal recessive	
PRICKLE1	608500	Epilepsy, progressive myoclonic 1B	Autosomal recessive	LOF
SNIP1	608241	Psychomotor retardation, epilepsy, and craniofacial dysmorphism	Autosomal recessive	LOF
CHD2	602119	Epileptic encephalopathy, childhood-onset	Autosomal dominant	LOF
ALDH7A1	107323	Epilepsy, pyridoxine-dependent	Autosomal recessive	LOF
PNPO	603287	Pyridoxamine 5'-phosphate oxidase deficiency	Autosomal recessive	LOF
WWOX	605131	Epileptic encephalopathy, early infantile, 28	Autosomal recessive	LOF
		Esophageal squamous cell carcinoma, somatic		
		Spinocrebellar ataxia, autosomal recessive 12	Autosomal recessive	
ALG13	300776	Epileptic encephalopathy, early infantile, 36	X-linked dominant	n/a
		Congenital disorder of glycosylation, type Is		
ASAH1	613468	Farber lipogranulomatosis	Autosomal recessive	LOF
		Spinal muscular atrophy with progressive myoclonic epilepsy	Autosomal recessive	LOF
CLN8	607837	Ceroid lipofuscinosis, neuronal, 8	Autosomal recessive	n/a
		Ceroid lipofuscinosis, neuronal, 8, Northern epilepsy variant	Autosomal recessive	

CDKL5	300203	Epileptic encephalopathy, early infantile, 2	X-linked dominant	LOF
EPM2A	607566	Epilepsy, progressive myoclonic 2A (Lafora)	Autosomal recessive	LOF
SIK1	605705	Epileptic encephalopathy, early infantile, 30	Autosomal dominant	n/a
STRADA	608626	Polyhydramnios, megalencephaly, and symptomatic epilepsy	Autosomal recessive	LOF
ARHGEF9	300429	Epileptic encephalopathy, early infantile, 8	X-linked recessive	LOF
DEPDC5	614191	Epilepsy, familial focal, with variable foci 1	Autosomal dominant	LOF
GNAO1	139311	Epileptic encephalopathy, early infantile, 17	Autosomal dominant	n/a
		Neurodevelopmental disorder with involuntary movements	Autosomal dominant	
PLCB1	607120	Epileptic encephalopathy, early infantile, 12	Autosomal recessive	LOF
TBC1D24	613577	Deafness, autosomal recessive 86	Autosomal recessive	
		Deafness, autosomal dominant 65	Autosomal dominant	
		DOOR syndrome	Autosomal recessive	LOF
		Epileptic encephalopathy, early infantile, 16	Autosomal recessive	LOF
		Myoclonic epilepsy, infantile, familial	Autosomal recessive	
CNTNAP2	604569	Cortical dysplasia-focal epilepsy syndrome	n/a	LOF
		Pitt-Hopkins like syndrome 1	n/a	
		{Autism susceptibility 15}	n/a	
LGI1	604619	Epilepsy, familial temporal lobe, 1	Autosomal dominant	LOF
PCDH19	300460	Epileptic encephalopathy, early infantile, 9	X-linked	LOF
RELN	600514	Lissencephaly 2 (Norman-Roberts type)	Autosomal recessive	LOF
		{Epilepsy, familial temporal lobe, 7}	Autosomal dominant	LOF
SRPX2	300642	(Rolandic epilepsy, mental retardation, and speech dyspraxia)	n/a	LOF
SZT2	615463	Epileptic encephalopathy, early infantile, 18	Autosomal recessive	LOF
PAFAH1B1	601545	Lissencephaly	Isolated cases	LOF
		Subcortical laminar heterotopia		
CPA6	609562	Epilepsy, familial temporal lobe, 5	Autosomal dominant; Autosomal recessive	LOF
		Febrile seizures, familial, 11		
CSTB	601145	Epilepsy, progressive myoclonic 1A (Unverricht and Lundborg syndrome)	Autosomal recessive	LOF
NHLRC1	608072	Epilepsy, progressive myoclonic 2B (Lafora)	Autosomal recessive	LOF
SCARB2	602257	Epilepsy, progressive myoclonic 4, with or without renal failure	Autosomal recessive	LOF
ST3GAL5	604402	Salt and pepper developmental regression syndrome	Autosomal recessive	LOF
ST3GAL3	138140	Autosomal Recessive Mental Retardation 12	Autosomal dominant	LOF
		Early Infantile Epileptic Encephalopathy 15		
KCNC1	616187	Epilepsy, progressive myoclonic 7	Autosomal dominant	n/a

Supplementary Table 2: The 40 genes targeted for the Epilepsy Zebrafish Project.

Human gene	Zebrafish gene	Protein Sequence Reference	% protein identity (GRCz10)	% DIOPT	Homology Score
ALDH7A1	*aldh7a1*	ENSDARP00000108190	81	75	78
ARHGEF9	*arhgef9a*	ENSDARP00000118893	79	100	90
	arhgef9b	ENSDARP00000115968	84	75	80
ARX	*arxa*	ENSDARP00000075256	68	75	72
	arxb	not identified			
CDKL5	*cdkl5*	ENSDARP00000111280	54	92	73
CHD2	*chd2*	ENSDARP00000108411	73	67	70
CNTNAP2	*cntnap2a*	(ENSDART00000178326.1)	71	67	69
	cntnap2b	ENSDARP00000104097	65	50	58
CPA6	*cpa6*	ENSDARP00000096966	64	83	74
DEPDC5	*depdc5*	ENSDARP00000098526	75	58	67
DNM1	*dnm1a*	ENSDARP00000124266	89	50	70
	dnm1b	ENSDARP00000088100	88	75	82
EEF1A2	*eef1a2*	ENSDARP00000010921	92	92	92
EPM2A	*epm2a*	ENSDARP00000132560	62	42	52
GABRA1	*gabra1*	ENSDARP00000090772	84	92	88
GABRB3	*gabrb3*	ENSDARP00000081734	73	83	78
GABRG2	*gabrg2*	ENSDARP00000087253	83	83	83
GNAO1	*gnao1a*	ENSDARP00000124476	90	92	91
	gnao1b	ENSDARP00000052345	84	58	71
GOSR2	*gosr2*	ENSDARP00000069524	69	83	76
GRIN1	*grin1a*	ENSDARP00000093144	88	75	82
	grin1b	ENSDARP00000038151	88	92	90
GRIN2A	*grin2aa*	ENSDARP00000116766	67	83	75
	grin2ab	not identified			
HNRNPU	*hnrnpua*	ENSDARP00000144487	52	75	64
	hnrnpub	ENSDARP00000112099	57	83	70
KCNA2	*kcna2a*	not identified			
	kcna2b	ENSDARP00000130579	92	83	88
KCNMA1	*kcnma1a*	ENSDARP00000118939	87	67	77
MEF2C	*mef2ca*	not identified			
	mef2cb	ENSDARP00000138296	74	75	75
PAFAH1B1	*pafah1b1a*	ENSDARP00000042217	94	92	93
	pafah1b1b	ENSDARP00000039257	93	92	92
PCDH19	*pcdh19*	ENSDARP00000124001	70	67	68
PLCB1	*plcb1*	not identified			
PNPO	*pnpo*	ENSDARP00000011179	64	75	70
PRICKLE1	*prickle1a*	ENSDARP00000059513	69	100	85

	prickle1b	not identified			
SCN1B	*scn1ba*	ENSDARP00000079066	36	100	68
SCN1A	*scn1laa*	ENSDARP00000138437	67	67	67
	scn1lab	ENSDARP00000125843	77	58	68
SCN8A	*scn8aa*	ENSDARP00000024690	83	83	83
	scn8ab	ENSDARP00000126281	84	75	80
SIK1	*sik1*	ENSDARP00000077468	57	75	66
SLC2A1	*slc2a1a*	ENSDARP00000022579	74	92	83
SLC6A1	*slc6a1a*	ENSDARP00000119658	73	83	78
	slc6a1b	ENSDARP00000005281	84	100	92
SPTAN1	*spna2*	ENSDARP00000093027	90	83	87
ST3GAL3	*st3gal3b*	ENSDARP00000110277	63	100	82
STRADA	*strada*	ENSDARP00000115217	70	67	68
STX1B	*stx1b*	ENSDARP00000076389	97	100	99
STXBP1	*stxbp1a*	ENSDARP00000012776	85	83	84
	stxbp1b	ENSDARP00000026241	77	67	72
SYNGAP1	*syngap1a*	ENSDARP00000144044	63	75	69
	syngap1b	ENSDARP00000087797	63	67	65
TBC1D24	*tbc1d24*	ENSDARP00000128484	55	83	69

Supplementary Table 3: Characterization of zebrafish orthologues for phenotypic characterization

Human	Zebrafish	Homology	Brain Expression	Development	Phenotypic Characterization
	scn1lab*				Y
SCN1A	_scn1laa_				
	scn8aa	_scn8aa_	_scn8aa_	_scn8aa_	Y
SCN8A	_scn8ab_			_scn8ab_	
GABRA1	_gabra1_	_gabra1_	_gabra1_	_gabra1_	Y
GABRB3	_gabrb3_	_gabrb3_	_gabrb3_	_gabrb3_	Y
GABRG2	_gabrg2_	_gabrg2_	_gabrg2_	_gabrg2_	Y
	grin2aa	_grin2aa_	_grin2aa_	_grin2aa_	No F3 generation
GRIN2A	_grin2ab_				
	slc2a1a	_slc2a1a_	_slc2a1a_	_slc2a1a_	Y
SLC2A1	_slc2a1b_				
	slc6a1a	_slc6a1a_	_slc6a1a_		No Cutting
SLC6A1	_slc6a1b_	_slc6a1b_	_slc6a1b_	_slc6a1b_	Y
	dnm1a	_dnm1a_	_dnm1a_	_dnm1a_	No F3 generation
DNM1	_dnm1b_				
	stxbp1a				
STXBP1	_stxbp1b*_				Y
	syngap1a	_syngap1a_	_syngap1a_	_syngap1a_	No F3 generation
SYNGAP1	_syngap1b_	_syngap1b_		_syngap1b_	Y
	arxa	_arxa_	_arxa_	_arxa_	Y
ARX	_arxb_				
EEF1A2	_eef1a2_	_eef1a2_	_eef1a2_	_eef1a2_	Y
	hnrnpua	_hnrnpua_	_hnrnpua_	_hnrnpua_	Y
HNRNPU	_hnrnpub_	_hnrnpub_	_hnrnpub_	_hnrnpub_	Y
	mef2ca				
MEF2C	_mef2cb_	_mef2cb_	_mef2cb_	_mef2cb_	Y
CHD2	_chd2_	_chd2_	_chd2_	_chd2_	Y
ALDH7A1	_aldh7a1_	_aldh7a1_	_aldh7a1_	_aldh7a1_	Y
PNPO	_pnpo_	_pnpo_	_pnpo_	_pnpo_	Y
CDKL5	_cdkl5_	_cdkl5_	_cdkl5_	_cdkl5_	Y
GNAO1	_gnao1a_	_gnao1a_	_gnao1a_	_gnao1a_	Y

	gnao1b	*gnao1b*	*gnao1b*	*gnao1b*	Y
TBC1D24	*tbc1d24*	*tbc1d24*	*tbc1d24*	*tbc1d24*	No F3 generation
PCDH19	*pcdh19*	*pcdh19*	*pcdh19*	*pcdh19*	Y
	pafah1b1a	*pafah1b1a*	*pafah1b1a*	*pafah1b1a*	Y
PAFAH1B1	*pafah1b1b*	*pafah1b1b*	*pafah1b1b*	*pafah1b1b*	Y
	kcna2a				
KCNA2	*kcna2b*	*kcna2b*	*kcna2b*	*kcna2b*	Y
KCNMA1	*kcnma1a*	*kcnma1a*			
	prickle1a	*prickle1a*		*prickle1a*	No F3 generation
PRICKLE1	*prickle1b*				
EPM2A	*epm2a*	*epm2a*	*epm2a*	*epm2a*	Y
SIK1	*sik1*	*sik1*	*sik1*	*sik1*	Y
	arhgef9a	*arhgef9a*	*arhgef9a*	*arhgef9a*	Y
ARHGEF9	*arhgef9b*	*arhgef9b*	*arhgef9b*	*arhgef9b*	Y
CPA6	*cpa6*	*cpa6*	*cpa6*	*cpa6*	Y
STRADA	*strada*	*strada*	*strada*	*strada*	Y
SPTAN1	*spna2*	*spna2*			
DEPDC5	*depdc5*	*depdc5*	*depdc5*	*depdc5*	Y
	cntnap2a	*cntnap2a*	*cntnap2a*	*cntnap2a*	Y
CNTNAP2	*cntnap2b*	*cntnap2b*	*cntnap2b*	*cntnap2b*	Y
	scn1ba	*scn1ba*	*scn1ba*	*scn1ba*	Y
SCN1B	*scn1bb*				
ST3GAL3	*st3gal3b*	*st3gal3b*	*st3gal3b*	*st3gal3b*	Y
PLCB1	*plcb1*	*plcb1*	*plcb1*	*plcb1*	No Cutting
GOSR2	*gosr2*	*gosr2*	*gosr2*	*gosr2*	No F3 generation
STX1B	*stx1b*	*stx1b*	*stx1b*	*stx1b*	No F3 generation
	grin1a	*grin1a*	*grin1a*	*grin1a*	Y
GRIN1	*grin1b*	*grin1b*	*grin1b*	*grin1b*	Y
**Total:**	**57**	**48**	**44**	**46**	

* indicate control genes with previously characterized seizure phenotypes.

Supplementary Figure 1| Local field potential recordings are minimally invasive. (**a**) Five dpf larvae were left freely swimming in embryo medium or subjected to agar embedding or agar embedding with electrode implantation, and behavior was tracked 4 hr and 24 hr after each treatment. Results show no significant differences in the total distance traveled (**b**) or maximum velocity of larvae when compared across the treatment groups. Data displayed as mean ± SEM.

Supplementary Figure 2| Distribution of ictal-like events. Histograms depict number and duration of ictal events cumulatively (**a-b**) and across individual EZP-epilepsy lines (**c-j**). Asterisk for *grin1b* designates heterozygote. Interictal events were measured using a custom MATLAB-based program for EZP-epilepsy lines and WT siblings.

Supplementary Figure 3| Kaplan-Meier survival curves for zebrafish CRISPR lines. Plots of survival for unfed WT, heterozygous and homozygous larvae across all zebrafish lines.

Supplementary Figure 4| Basal locomotor activity of epileptic zebrafish lines. Five dpf larval zebrafish were tracked in the behavioral assay and graphs depict (**a**) total distance traveled, **(b)** maximum velocity, (**c**) number of high-speed events (HSE) and (**d**) number of long duration HSE observed across the various lines. Total distance and maximum velocity were extracted directly from EthoVision XT 11.5 software while the number of events ≥ 28 mm/s (HSE) and long duration HSE (≥1 s) were scored using an in-house MATLAB algorithm. Data displayed as scatter plots showing individual larval values and error bars represent mean and SEM. Statistics calculated using One-way ANOVA and *post hoc* Dunnett multiple comparison test, *$p \leq 0.05$, **$p \leq 0.005$, **$p < 0.0001$. N values are provided in the plots next to each larvae type.

Supplementary Figure 5| Wild-type recording. Representative Type 0 raw LFP recording trace along with a corresponding wavelet time-frequency spectrogram for a representative WT zebrafish larvae. Scale bar = 500 μV.

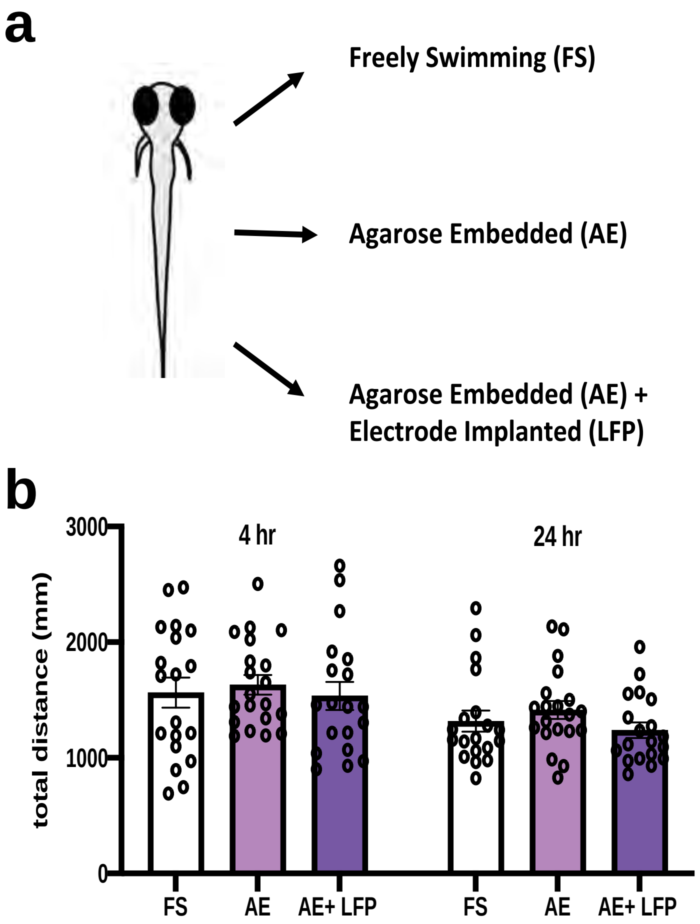
a
Freely Swimming (FS)
Agarose Embedded (AE)
Agarose Embedded (AE) +
Electrode Implanted (LFP)
b
4 hr
24 hr
total distance (mm)
3000
2000
1000
0
FS
AE
AE+ LFP
FS
AE
AE+ LFP

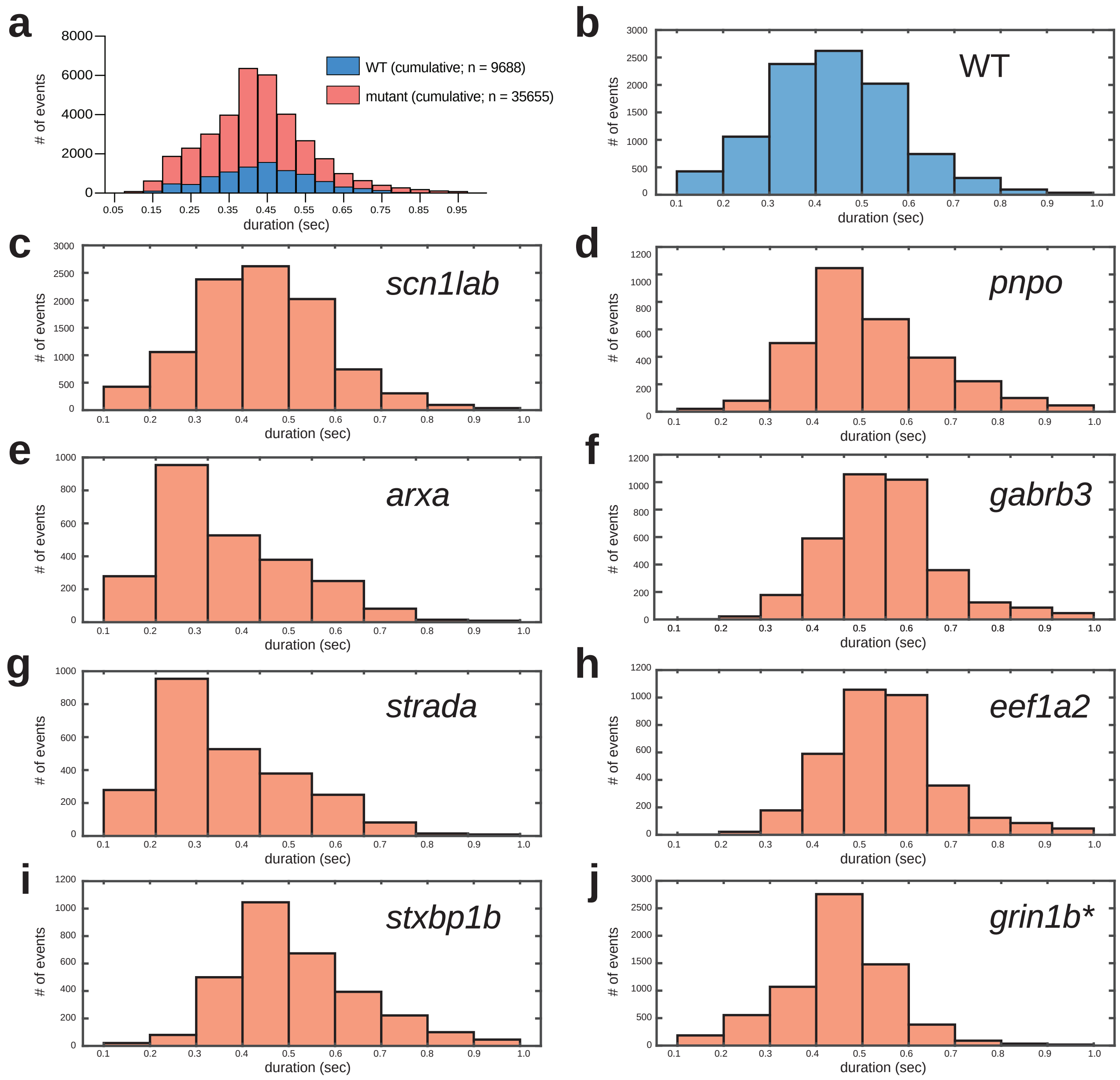
a
b
c
d
e
f
g
h
i
j
WT (cumulative; n = 9688)
mutant (cumulative; n = 35655)
# of events
duration (sec)
WT
scn1lab
pnpo
arxa
gabrb3
strada
eef1a2
stxbp1b
grin1b*

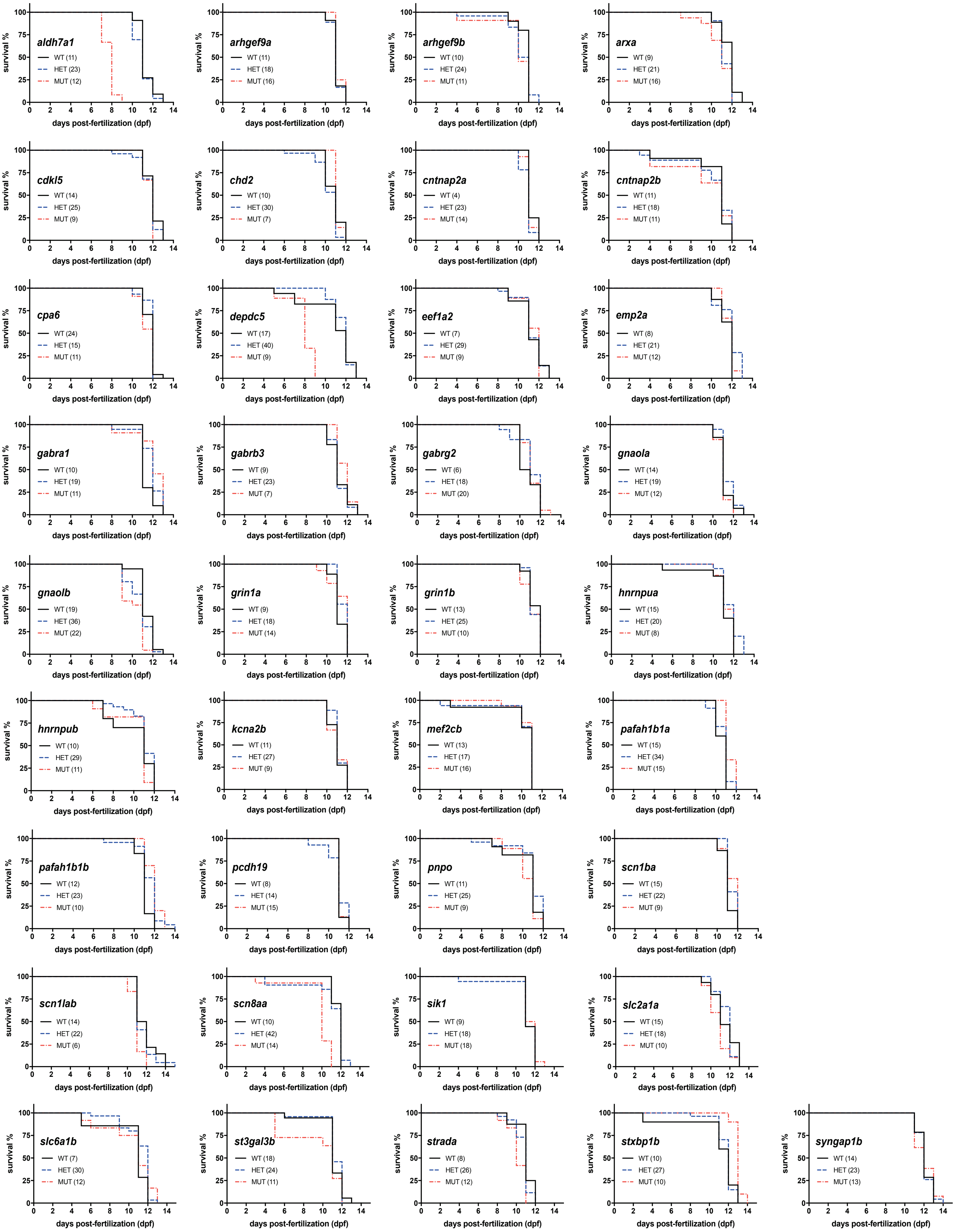
survival %
days post-fertilization (dpf)
aldh7a1
WT (11)
HET (23)
MUT (12)
arhgef9a
WT (11)
HET (18)
MUT (16)
arhgef9b
WT (10)
HET (24)
MUT (11)
arxa
WT (9)
HET (21)
MUT (16)
cdkl5
WT (14)
HET (25)
MUT (9)
chd2
WT (10)
HET (30)
MUT (7)
cntnap2a
WT (4)
HET (23)
MUT (14)
cntnap2b
WT (11)
HET (18)
MUT (11)
cpa6
WT (24)
HET (15)
MUT (11)
depdc5
WT (17)
HET (40)
MUT (9)
eef1a2
WT (7)
HET (29)
MUT (9)
emp2a
WT (8)
HET (21)
MUT (12)
gabra1
WT (10)
HET (19)
MUT (11)
gabrb3
WT (9)
HET (23)
MUT (7)
gabrg2
WT (6)
HET (18)
MUT (20)
gnaola
WT (14)
HET (19)
MUT (12)
gnaolb
WT (19)
HET (36)
MUT (22)
grin1a
WT (9)
HET (18)
MUT (14)
grin1b
WT (13)
HET (25)
MUT (10)
hnrnpua
WT (15)
HET (20)
MUT (8)
hnrnpub
WT (10)
HET (29)
MUT (11)
kcna2b
WT (11)
HET (27)
MUT (9)
mef2cb
WT (13)
HET (17)
MUT (16)
pafah1b1a
WT (15)
HET (34)
MUT (15)
pafah1b1b
WT (12)
HET (23)
MUT (10)
pcdh19
WT (8)
HET (14)
MUT (15)
pnpo
WT (11)
HET (25)
MUT (9)
scn1ba
WT (15)
HET (22)
MUT (9)
scn1lab
WT (14)
HET (22)
MUT (6)
scn8aa
WT (10)
HET (42)
MUT (14)
sik1
WT (9)
HET (18)
MUT (18)
slc2a1a
WT (15)
HET (18)
MUT (10)
slc6a1b
WT (7)
HET (30)
MUT (12)
st3gal3b
WT (18)
HET (24)
MUT (11)
strada
WT (8)
HET (26)
MUT (12)
stxbp1b
WT (10)
HET (27)
MUT (10)
syngap1b
WT (14)
HET (23)
MUT (13)

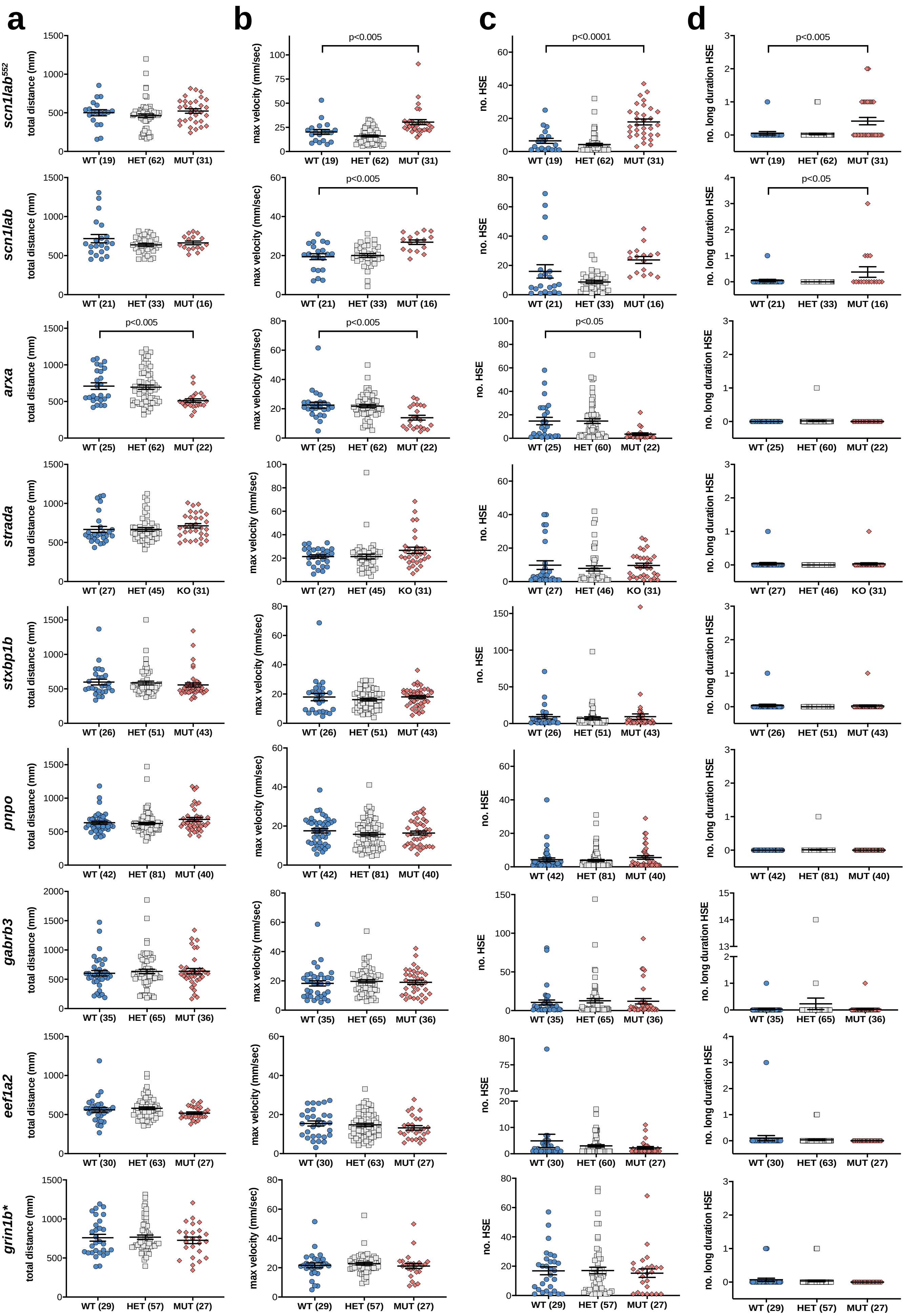

a
b
c
d
scn1lab552
scn1lab
arxa
strada
stxbp1b
pnpo
gabrb3
eef1a2
grin1b*
total distance (mm)
max velocity (mm/sec)
no. HSE
no. long duration HSE
p<0.005
p<0.0001
p<0.05
WT (19) HET (62) MUT (31)
WT (21) HET (33) MUT (16)
WT (25) HET (62) MUT (22)
WT (25) HET (60) MUT (22)
WT (27) HET (45) KO (31)
WT (27) HET (46) KO (31)
WT (26) HET (51) MUT (43)
WT (42) HET (81) MUT (40)
WT (35) HET (65) MUT (36)
WT (30) HET (63) MUT (27)
WT (30) HET (60) MUT (27)
WT (29) HET (57) MUT (27)

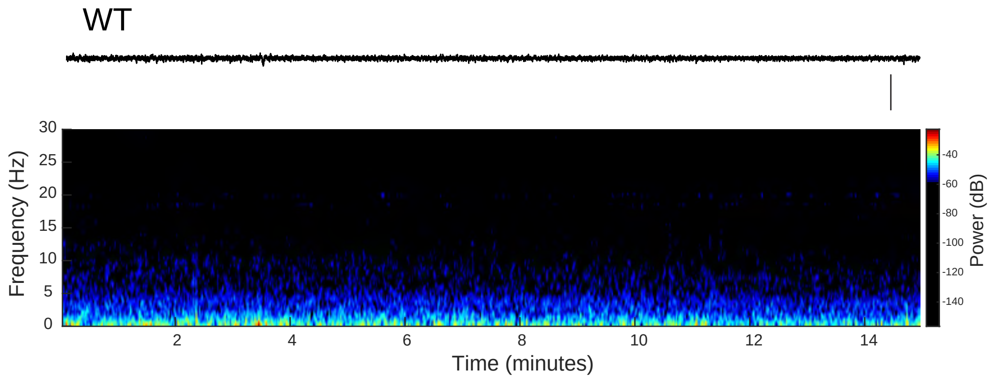

WT
Frequency (Hz)
30
25
20
15
10
5
0
2
4
6
8
10
12
14
Time (minutes)
-40
-60
-80
-100
-120
-140
Power (dB)
