# Supplementary material for: Phenotypic analysis of catastrophic childhood epilepsy genes
Source: Commun Biol. 2021 Jun 3;4:680. doi: 10.1038/s42003-021-02221-y (PMC8175701; doi:10.1038/s42003-021-02221-y)
Supplement: Supplementary file 2 — Reporting Summary [file 42003_2021_2221_MOESM2_ESM.pdf]

nature research

Corresponding author(s): *Double-blind peer review submissions: write DBPR and your manuscript number here instead of author names.*

Last updated by author(s): *YYYY-MM-DD*

Reporting Summary

Nature Research wishes to improve the reproducibility of the work that we publish. This form provides structure for consistency and transparency in reporting. For further information on Nature Research policies, see our Editorial Policies and the Editorial Policy Checklist.

Statistics

For all statistical analyses, confirm that the following items are present in the figure legend, table legend, main text, or Methods section.

n/a	Confirmed	
☐	☒	The exact sample size (*n*) for each experimental group/condition, given as a discrete number and unit of measurement
☒	☐	A statement on whether measurements were taken from distinct samples or whether the same sample was measured repeatedly
☐	☒	The statistical test(s) used AND whether they are one- or two-sided<br>*Only common tests should be described solely by name; describe more complex techniques in the Methods section.*
☒	☐	A description of all covariates tested
☐	☒	A description of any assumptions or corrections, such as tests of normality and adjustment for multiple comparisons
☐	☒	A full description of the statistical parameters including central tendency (e.g. means) or other basic estimates (e.g. regression coefficient) AND variation (e.g. standard deviation) or associated estimates of uncertainty (e.g. confidence intervals)
☐	☒	For null hypothesis testing, the test statistic (e.g. *F*, *t*, *r*) with confidence intervals, effect sizes, degrees of freedom and *P* value noted<br>*Give P values as exact values whenever suitable.*
☒	☐	For Bayesian analysis, information on the choice of priors and Markov chain Monte Carlo settings
☒	☐	For hierarchical and complex designs, identification of the appropriate level for tests and full reporting of outcomes
☒	☐	Estimates of effect sizes (e.g. Cohen's *d*, Pearson's *r*), indicating how they were calculated

Our web collection on statistics for biologists contains articles on many of the points above.

Software and code

Policy information about availability of computer code

Data collection	Axoscope and Clampfit software was used for acquisition and analysis of electrophysiology data. EthoVision software was used for acquisition and analysis of behavioral data. ImageJ was used for analysis of imaging data. GraphPad Prism was used for analysis. MATLAB was used to develop customized data analysis. All of these data collection and analysis modes are indicated in the manuscript.
Data analysis	See statement above. A statement that MATLAB code is available upon reasonable request is included in the Methods section.

For manuscripts utilizing custom algorithms or software that are central to the research but not yet described in published literature, software must be made available to editors and reviewers. We strongly encourage code deposition in a community repository (e.g. GitHub). See the Nature Research guidelines for submitting code & software for further information.

Data

Policy information about availability of data

All manuscripts must include a data availability statement. This statement should provide the following information, where applicable:

- Accession codes, unique identifiers, or web links for publicly available datasets
- A list of figures that have associated raw data
- A description of any restrictions on data availability

All custom MATLAB programs will be made available upon reasonable request. Representative electrophysiology tracings, Kaplan-Meier survival plots, behavioral data, sequencing information are available on our web-portal (https://zebrafishproject.ucsf.edu). The datasets generated during the current studies are available from the corresponding author on reasonable request.

Field-specific reporting

Please select the one below that is the best fit for your research. If you are not sure, read the appropriate sections before making your selection.

☒ Life sciences ☐ Behavioural & social sciences ☐ Ecological, evolutionary & environmental sciences

For a reference copy of the document with all sections, see nature.com/documents/nr-reporting-summary-flat.pdf

Life sciences study design

All studies must disclose on these points even when the disclosure is negative.

Sample size	Using mutant (scn1lab) and WT control data sets from prior studies, we made a sample-size calculation with a power of 99.9% and p = 0.001 to minimize false positives. This analysis determined that a sample size of 16 will be sufficient to detect a 60% effect in mutant larvae relative to WT controls or baseline.
Data exclusions	No data was excluded.
Replication	All experiments included biological replicates on separate clutches of larvae. All experiments were repeated in triplicate.
Randomization	Larvae used for all experiments were distributed to different behavioral, electrophysiological or imaging protocols using a block randomization protocol. The block randomization method is designed to randomize subjects into groups that result in equal sample sizes and ensures a balance in sample size across groups over time. The block size chosen here is based on prior studies and is a multiple of the number of groups i.e., two experimental groups, WT and homozygous mutant . We used a total block size of n = 30 randomly chosen larvae to obtain a minimum of 6-8 homozygous mutants per experiment.
Blinding	To eliminate any potential investigator bias, all experiments incorporated (i) blinded conduct as zebrafish caretakers and investigators executing experiments are blinded to the identity of the zebrafish breeding pair and (ii) blinded assessment of outcome as all measurement or quantification of experimental outcomes are performed on coded files by independent investigators. All larvae were independently tested post hoc by extracting DNA from each fish and genotyping the appropriate gene locus. To achieve this level of rigor, lab technicians are solely responsible for breeding and maintenance of all zebrafish lines, graduate students or postdoctoral fellows perform experiments in a blinded manner and code the resulting data files, and finally a different lab technician, graduate student, postdoc or PI analyzes the coded data files.

Reporting for specific materials, systems and methods

We require information from authors about some types of materials, experimental systems and methods used in many studies. Here, indicate whether each material, system or method listed is relevant to your study. If you are not sure if a list item applies to your research, read the appropriate section before selecting a response.

Materials & experimental systems

n/a	Involved in the study	
☒	☐	Antibodies
☒	☐	Eukaryotic cell lines
☒	☐	Palaeontology and archaeology
☐	☒	Animals and other organisms
☒	☐	Human research participants
☒	☐	Clinical data
☒	☐	Dual use research of concern

Methods

n/a	Involved in the study	
☒	☐	ChIP-seq
☒	☐	Flow cytometry
☒	☐	MRI-based neuroimaging

Animals and other organisms

Policy information about studies involving animals; ARRIVE guidelines recommended for reporting animal research

Laboratory animals	Zebrafish larvae were used for all experiments. At this stage larvae are sexually indistinguishable.
Wild animals	N/A
Field-collected samples	N/A
Ethics oversight	All procedures described herein were performed in accordance with the Guide for the Care and Use of Animals (ebrary Inc., 2011) and adhered to guidelines approved by the University of California, San Francisco Institution Animal Care and Use Committee (IACUC approval #: AN171512-03A).

Note that full information on the approval of the study protocol must also be provided in the manuscript.
